# Seroprevalence and risk factors associated with hepatitis C: a cross-sectional study of persons who inject drugs in Puerto Rico, 2018

**DOI:** 10.1186/s12889-023-15341-3

**Published:** 2023-04-18

**Authors:** Vivian Colón-López, Paola M. Alvelo-Fernández, Nadia Centeno-Alvarado, Ivony Y. Agudelo Salas, Yadira Rolón Colón, María Pabón Martínez, Jorge L. Rodríguez-Lebrón, Juan C. Reyes-Pulliza

**Affiliations:** 1 Cancer Control and Population Sciences Program, University of Puerto Rico Comprehensive Cancer Center, Primer Piso Oficina #151 Paseo José C. Barbosa San Juan, San Juan, 00935 Puerto Rico; 2 Graduate School of Public Health, Medical Sciences Campus, University of Puerto Rico, San Juan, Puerto Rico; 3 HIV/AIDS Surveillance Program, Puerto Rico Department of Health, San Juan, Puerto Rico; 4 Department of Biostatistics and Epidemiology, Graduate School of Public Health, Medical Sciences Campus, University of Puerto Rico, San Juan, Puerto Rico

**Keywords:** HIV, NHBS, Puerto Rico, PWID, HCV

## Abstract

**Background:**

People Who Inject Drugs (PWID) are at a higher risk of acquiring bloodborne infections. We aimed to estimate the seroprevalence of the Hepatitis C Virus (HCV) in PWID and identify correlates and risk factors using data from the Puerto Rico National HIV Behavioral Surveillance System, PWID cycle 5, conducted in 2018.

**Methods:**

A total of 502 San Juan Metropolitan Statistical Area participants were recruited through the Respondent Driven Sampling method. Sociodemographic, health-related, and behavioral characteristics were assessed. Testing for HCV antibodies was completed after the face-to-face survey. Descriptive and logistic regression analyses were performed.

**Results:**

Overall seroprevalence of HCV was 76.5% (95% CI: 70.8-81.4%). A significantly (p < 0.05) higher HCV seroprevalence was observed among PWID with the following characteristics: heterosexuals (78.5%), high school graduates (81.3%), tested for sexually transmitted infections (STI) in the past 12 months (86.1%), frequent speedball injection (79.4%), and knowing the HCV serostatus of the last sharing partner (95.4%). Adjusted logistic regression models showed that having completed high school and reported STI testing in the past 12 months were significantly associated with HCV infection (OR_a_ = 2.23; 95% CI: 1.06–4.69; OR_a_ = 2.14; 95% CI: 1.06–4.30, respectively).

**Conclusions:**

We report a high seroprevalence of HCV infection in PWID. Social health disparities and potential missed opportunities validate the continuing call for local action for public health and prevention strategies.

## Background

It is well documented that People Who Inject Drugs (PWID) are at a higher risk of acquiring various infections through blood, such as Human Immunodeficiency Virus (HIV) and Hepatitis C Virus (HCV) [1–3]. There are nearly 11 million PWID worldwide, a group that continues to be impacted by the HIV/AIDS epidemic with an estimated 1.4 million PWID living with HIV [4]. In Puerto Rico (PR), from 1981 to 1999, PWID accounted for most cases of HIV [5]. Data from the PR HIV Surveillance System shows that in 2010, more than 20% of new HIV infections were diagnosed among PWID [6].

Like HIV, HCV in PWID is a public health concern with potential chronic severe sequelae if left unattended. Worldwide, around 5.5 million PWID live with Hepatitis C [4]. In PR, an epidemiological study conducted among PWID in an urban setting reported a prevalence of HCV of 89% [7]; and 78.4% among PWID from rural towns [8]. Like other studies, risk behaviors such as sharing injection equipment [8], injecting more than seven times a day and reusing syringes [9], have been associated with HCV infection.

The National HIV Behavioral Surveillance System (NHBS) has identified an increase in age among cohorts recruited as part of this surveillance system [10]. HCV can increase the progression of liver damage, the risk of highly active antiretroviral therapy-related hepatotoxicity, and mortality in people living with HIV [11, 12]. In addition, chronic infection with HIV coinfection has been identified as a risk factor for hepatocellular cancer, the most common type of primary liver cancer [13, 14]. The biological interaction between HCV and HIV, particularly in older cohorts, is worrisome, given that PR has been characterized by consistently having one of the highest incidence rates of HIV/AIDS in all states and territories of the United States (US) [11].

Although the epidemiology of HIV and HCV among PWID has been extensively evaluated across different studies in PR [7, 8, 11, 15–19], there has been no up-to-date study to assess the burden of HCV and emergent practices among PWID in PR. We thus aim to estimate the HCV seroprevalence in PWID. We also aim to identify correlates and risk factors associated with HCV using the PR National HIV Behavioral Surveillance system PWID cycle five conducted in 2018 (PR-NHBS-PWID-5). We estimate HCV seroprevalence using NHBS-PWID data in PR for the first time. The value of the data collected during the chosen cycle is that before this assessment, HCV prevalence using NHBS data relied only on self-report. Having more recent HCV estimates will help us delineate future HCV prevention efforts in PR.

## Methods

### Data source

#### National HIV behavioral surveillance study

NHBS is a serial cross-sectional survey that monitors the prevalence of HIV, sexual and drug-use behaviors, and HIV testing. The methodology used in the NHBS study has been described elsewhere [20, 21]. We used data from the fifth PWID cycle of the PR-NHBS completed in 2018. A total of 506 participants were part of the study. Briefly, the NHBS-PWID-5 (2018) recruitment was done through the Respondent Driven Sampling method (RDS), a chain-referral sampling strategy similar to snowball sampling that allows reaching hidden populations through a dual incentive system [22, 23]. Respondent Driven Sampling begins with the selection of a small number of recruiters (seeds) by the NHBS staff after a formative assessment with key informants who work with PWID. These seeds recruit project participants, who recruit other participants forming a chain [24]. Our analysis included data from PWID participants (18 years of age or older) who reported injecting drugs during the previous 12 months. Recent needle track marks were confirmed by the NHBS staff. Other inclusion criteria included: current residents of the San Juan Metropolitan Statistical Area (SJ-MSA) who could complete the interview in English or Spanish. Participants were recruited in community centers that provide services to the PWID population.

### Procedures

Participants signed informed consent before beginning the survey, which was read by an interviewer who also offered a copy to the participants for their record. Recruited participants completed an anonymous standardized face-to-face survey that lasted approximately 25 min with trained interviewers who noted responses using a handheld computer device with the Questionnaire Development System software (NOVA Research Company, version 2.6.1, USA). Both HIV and HCV tests were offered to those who completed the interview. The interview included questions about demographics, sexual practices, drug use, and HIV and HCV testing. The participant’s responses and test results were labeled with a survey number, removing identifying information.

### Measures

We used the following variables from the NHBS core questionnaire for our analysis [25]: age, gender, sexual orientation, educational attainment, household income, and health insurance coverage. Characteristics and behaviors of possible exposure to HCV included HIV serostatus and injection practices such as age at first injection and frequency of injection of drugs. Other characteristics, such as awareness of the last sharing partner’s HCV serostatus, were assessed in the questionnaire.

#### Detection of antibodies to hepatitis C virus

Testing for HCV was done using the OraQuick® HCV Rapid Antibody Test (OraSure Technologies Inc., Bethlehem, PA, USA) by following the manufacturer’s instructions. The test is an immunoassay for the qualitative detection of antibodies to the HCV (anti-HCV) and was performed through fingerstick and venipuncture of whole blood specimens from individuals 18 years or older. Antibodies in the blood reacting with these peptides and antigens are visualized by colloidal gold-labeled, generating a visible line in the test zone for a reactive sample. The PR-NHBS staff interpreted the result according to the manufacturer’s instructions 20 min later. The test has a sensitivity and specificity of 97.9% and 98.5%, respectively. In addition, for participants who tested positive on the rapid test, the RT-PCR Qualitative HCV RNA test was used as a confirmatory test with a sensitivity of 94.1%, positive predictive value of 72.7%, negative predictive value of 99.9% [26]; and specificity of 99.5 to 100% [27–29].

#### Detection of antigens and antibodies to HIV

Participants samples were tested using Alere Determine HIV-1/2 Ag/Ab combo assay (Abbott Diagnostics Scarborough, Inc., ME, USA), an immunochromatographic fingerstick test for the simultaneous and separate qualitative detection of free Human Immunodeficiency Virus Type 1 (HIV-1), p24 antigen, and antibodies to HIV-1 and HIV-2 in whole blood or plasma samples. The viral capsid protein p24 (core) present in HIV-1 and HIV-2 is detected in the blood before the HIV antibody during acute infection. The result was interpreted according to the manufacturer’s instructions between 20 and 30 min after the test. If the preliminary result was positive, we did the confirmatory test by another rapid test, INSTI® HIV-1/HIV-2 Antibody Test (bioLytical Laboratories Inc., Canada). INSTI® HIV-1/HIV-2 Antibody Test [30]; was used to detect antibodies to HIV-1 and HIV-2 in human whole blood, fingerstick blood, serum, or plasma providing test results in as little as 60 s. The device was spotted with HIV-1 and HIV-2 recombinant proteins, which react with HIV antibodies in the specimen. The procedure and the test reading were carried out following the manufacturer’s instructions. The test has a sensitivity and specificity > 99% [30].

### Statistical analysis

Participants identified as transgender (n = 4) were excluded from the sample due to the small sample size. Therefore, a total of 502 participants were included in our analysis. The data were weighted for HCV serostatus using the CDC software Respondent-driven Sampling Analysis Tool (RDSAT 8.1.45™). Descriptive analyses were stratified by HCV serostatus. HCV seroprevalence was calculated for sociodemographic, behavioral, and clinical characteristics. Chi-square and Fisher exact tests were used to assess statistical differences in the distribution of different characteristics and behaviors. A logistic regression analysis was used to assess the associations of selected variables that were statistically significant (p < 0.05) or tended to statistical significance (0.05 ≤ *p* < 0.10) in the bivariate analysis. Variables such as speedball (mixture of cocaine and heroin) injection frequency and the participant’s knowledge of the HCV serostatus of the last person with whom they shared injection equipment were significant in the bivariate analysis but were excluded in the final model because both are conditioned variables. We used behavioral and clinical data from the core PR-NHBS interview to describe drug use by HCV serostatus. Statistical significance was set at *p* < 0.05. All analyses were conducted using STATA 17™.

## Results

The sociodemographic, health-related, and drug consumption characteristics by HCV serostatus of the PWID are shown in Table 1. Most PWIDs were men (84.7%) and 40 years or older (66.5%). Most participants reported a household income ranging from $0 to $4,999 (81.4%). Moreover, 40.5% and 37.5% PWID reported completing less than high school or obtaining a high school diploma, respectively. More than half of PWID reported having health insurance (63.1%), with the majority being government insurance (98.4%). The weighted prevalence of HIV among the PWID was 10.8%. Most participants reported not having an STI test in the past 12 months before the interview (78.8%) *(data not shown)*.

The PWID weighted seroprevalence of HCV was 76.5%. Those PWID who reported being heterosexuals had a significantly higher HCV seroprevalence (78.5%). Those PWID who reported either being a high school graduate or having less than high school had a higher seroprevalence of HCV (81.3%), and 77.9%, respectively). The PWID who tested for STIs in the past 12 months had a significantly higher HCV seroprevalence (86.1%).

Of those PWID who started injecting at or before 20 years of age, 80.9% were HCV positive. Among PWID who reported injecting speedball daily or more than daily, 79.4% were HCV positive (Table 1). No statistical differences were noted regarding sharing behaviors among PWID but participants reported using a needle after someone else had used it the last time they shared with someone; and of these, 78.2% were HCV-positive. Meanwhile, 198 PWID reported using equipment (cooker, cotton, or water) after someone else had already used it; of those, 86.2% were HCV-positive (Table 1).

**Table 1:** Sociodemographic, health-related, and drug consumption characteristics by HCV Serostatus, PWID-NHBS Cycle 2018 (n = 502)

Characteristics	Total PWID sampleª	Unweighted Number^b^ of HCV+	Weighted Serostatus^c^		
			HCV+	HCV-	*p* value
**Total**	502	364	76.5	23.5	
**Gender**					
Male	425	311	75.5	24.5	0.40
Female	77	53	82.0	18.0	
Sexual orientation* (n = 501)					
Heterosexual	439	324	78.5	21.5	0.03
Homosexual or bisexual	62	40	59.9	40.1	
**Age**					
18–29	34	15	63.1	36.9	0.36
30–39	134	103	81.0	19.0	
40 years old or more	334	246	77.1	22.9	
Educational attainment** (n = 501)					
Less than high school	203	150	77.9	22.1	0.05
High school graduate	188	139	81.3	18.7	
Some college or more	110	75	64.1	35.9	
**Currently insured**					
No	185	130	71.2	28.8	0.14
Yes	317	234	79.5	20.5	
**Household income (n = 499)**					
$0-$4,999	406	301	78.0	22.0	0.43
$5,000–14,999	76	51	70.8	29.2	
$15,000 or more	17	10	64.7	35.3	
**STI** ^**d**^ testing in the past 12 months (n = 501)*					
No	395	283	73.1	26.9	0.03
Yes	106	80	86.1	13.9	
**HIV serostatus**					
Negative	446	318	76.0	24.0	0.65
Positive	56	46	80.4	19.6	
**Age at first injection† (n = 501)**					
≤ 20 years	263	205	80.9	19.1	0.08
> 20 years	238	158	71.7	28.3	
**Injection frequency (past 12 months)**					
≥ daily	474	343	76.5	23.5	0.98
< daily	28	21	76.7	23.3	
Speedball frequency* (n = 429)					
≥ daily	384	291	79.4	20.6	0.01
< daily	45	30	55.9	44.1	
**Sharing needles** ^**e**^ **(n = 294)**					
No	221	178	86.1	13.9	0.16
Yes	73	55	78.2	21.8	
**Sharing injection equipment** ^**f**^ **(n = 227)**					
No	29	22	77.0	23.0	0.25
Yes	198	159	86.2	13.8	
Knew HCV serostatus of last sharing person* (n = 295)					
No	232	177	81.0	19.0	0.001
Yes	63	57	95.4	4.6	

* *p* < 0.05 significance in weighted statistical analysis

** *p* = 0.05 significance in weighted statistical analysis

† 0.05 ≤ *p* < 0.10 tendency to significance in weighted statistical analysis

^a^ N = 502

^b^ Numbers might not add to the total because of missing or unknown data

^c^ Percentages are row percentages

^d^ Gonorrhea, chlamydia, or syphilis

^e^ Last time that shared used a needle after someone else had already injected with it

^f^ Last time that shared used a cooker, cotton, or water that someone else had already used

Adjusted weighted logistic regression analysis showed that PWID who had completed high school had increased odds of having HCV compared to those who had completed some college or more (OR_a_ = 2.23; 95% CI: 1.06–4.69). PWID tested for a sexually transmitted infection, such as gonorrhea, chlamydia, or syphilis, had higher odds of having HCV when compared to those that were not tested (OR_a_ = 2.14; 95% CI: 1.06–4.30). Moreover, those PWID who started injecting at or before 20 years of age had higher odds of being HCV positive when compared to those that started injecting at 21 years or more (OR_a_ = 1.46; 95% CI: 0.84–2.53); however, the relative excess was not statistically significant (p > 0.05) (Table 2).

**Table 2:** Weighted multivariate analysis for factors associated with HCV-positive serostatus among PWID, NHBS Cycle 2018 (n = 498)

**Characteristics**	**Crude OR ** **[95% CI]**	**Adjusted OR** ^**‡**^ **[95% CI]**	
Sexual Orientation*			
Homosexual/Bisexual	Reference	Reference	
Heterosexual	2.43 [1.08–5.51]	2.71 [1.21–6.09]	
**Education**			
Some college or more	Reference	Reference	
High school diploma*	2.43 [1.11–5.32]	2.23 [1.06–4.69]	
Less than high school	1.97 [0.92–4.21]	1.85 [0.89–3.87]	
STI testing in the past 12 months*			
No	Reference	Reference	
Yes	2.28 [1.09–4.76]	2.14 [1.06–4.30]	
**Age at first injection**			
> 20 years	Reference	Reference	
≤ 20 years	1.67 [0.93-3.00]	1.46 [0.84–2.53]	

* *p* < 0.05 significance in adjusted weighted statistical analysis

^‡^Adjusted for sexual orientation, education, STI testing, age at first injection, and gender

## Discussion

We describe the seroprevalence and correlates associated with HCV infection in a sample of PWID in the SJ-MSA of PR. A weighted HCV seroprevalence of 76.5% was observed. The HCV seroprevalence reported in our study is similar (78.4%) to a study of PWID in rural PR between April and June 2015 [8]; but lower (89%) than a study conducted a decade earlier among PWID living in the San Juan metropolitan area [7]. As expected, the prevalence observed in a population-based study was lower (6.3%) [31]. In addition to the high HCV seroprevalence observed, our results showed that a great number of participants share injection equipment, such as cotton, cookers, or water, which has been repeatedly found to be a risk behavior for HCV transmission [8, 32].

We also report that despite HCV high-risk behavioral practices identified, the practice of sharing needles was lower compared to other injection equipment use, which suggests harm reduction awareness within this group. Harm reduction programs have helped reduce the negative effects or consequences of certain behaviors, particularly drug use [33]. Locally, a few of these programs have been implemented, including *Punto Fijo* in the San Juan Metropolitan Area [34], *Intercambios Puerto Rico* in the Eastern region [35] and *El Punto en la Montaña* in the Central Eastern region [36]. These nonprofit community-based organizations provide new syringes and needles in exchange for used ones (syringe exchange programs) and harm reduction injection kits to reduce HIV/AIDS and Hepatitis transmission, which may have helped reduce the practice among PWID in the metropolitan area of San Juan [7].

In addition to sharing injection equipment, the lack of accessible HCV treatment in PR during the study period may have contributed to the high HCV prevalence. Most participants reported a household income of less than $5,000, which is lower than the estimated median household income [37]. Social disparities, such as lower income, can lead to limited access to treatment [38]. Treatment for HCV is expensive, which makes it less accessible for this population [8, 39, 40]. In recent years, a program offering free oral HCV treatment to those who are members of the PR government health insurance was established [41, 42]. However, awareness and accessibility of the program targeted to underserved and vulnerable populations such as PWID is limited [41, 42].

After adjusting for behavioral and hepatitis-related factors, lower education was associated with a high risk of HCV compared with the highest educational attainment group, which was also reported in a population-based study in PR [31]; and a Danish national registry [43]. Social health disparities, including lower education and socioeconomic status, directly and indirectly, influence PWID’s health [38]. Such disparities can lead to limited access to health resources, risk-reduction information and preventive care, and poorer quality of care, leading to a higher risk of infectious diseases such as HIV and HCV [38].

We report that testing for STIs in the past 12 months is positively associated with HCV which suggests that STI testing is a proxy for access to care, leading to HCV testing opportunities. Interventions by community-based organizations have helped identify HCV-positive cases since HIV/STI, and HCV testing opportunities or referrals to testing are made available to PWID [34–36, 44, 45]. Such community-based interventions may provide a feasible venue for further HCV testing and follow-up to determine chronic infection, genotype, and appropriate treatment, helping reduce the chronic impact of HCV among PWID in PR.

Frequent speedball injectors had a higher HCV prevalence. Studies have found that injection of speedball was associated with HCV infection [46, 47]. The injection of speedball can encourage the practice of pooling resources such as money or syringes to divide the mixture into smaller quantities [48], also known as backloading or obtaining drugs [8]. Our findings also showed that most participants who engaged in sharing behaviors were unaware of the HIV or HCV serostatus of the last person they shared with. Those PWID who use speedball daily or more are significantly more likely to inject frequently any drug (*p* < 0.001), heroin (*p* = 0.051), and painkillers (*p* = 0.064) and have lower education (*p* = 0.083) (post hoc analysis), which have been documented in other studies [49, 50].

We report that those who knew the HCV serostatus of the last sharing person had a higher HCV prevalence which could be explained by selecting higher risk partners based on concordant HCV serostatus [18]. Awareness of ones or sharing partner’s HCV serostatus does not necessarily imply positive changes in behaviors or decisions related to injection drug use if HCV is not considered a risk or concern [51, 52]. However, participants who perceived their partner was HCV positive were associated with decreased odds of engaging in receptive needle/syringe sharing [53]. Moreover, another study reported that PWID aware of their HIV-negative serostatus tended to inject drugs more safely [54], while those PWID who knew they were HIV positive were more likely to never give their drug equipment to other PWID [54].

### Limitations

Our study does have several limitations. Firstly, our participants of PWID in PR is mostly adults, which is similar to national data [55], it is important to establish a comparative analysis of adult and young PWID in PR to characterize their drug use practices as well as their behavioral practices [46]. Secondly, our study sample may not be representative of the entire PWID population in PR which may not enable generalization to other cohorts of PWID in PR. Thirdly, a few variables had missing values, which may have produced biased estimates. However, the range of missing values for the variables that were not conditioned was minimal. Fourthly, a chain-referral sampling like RDS has associated biases; however, RDS can control for such biases through proper data collection and analysis. Fifthly, there is the possibility of recall and self-desirability bias due to the participants being asked questions regarding behaviors in the past 12 months, which relies on self-reported data. Lastly, our methodology did not allow for establishing HCV temporal chronicity beyond the test results since the NHBS is a cross-sectional study, the impact of some characteristics associated with HCV cannot be assessed over time.

## Conclusion

We have shown that the burden of HCV in the PWID population in PR is high which demonstrates a challenge in health management due to pressing problems related to socioeconomic determinants of health. Efforts to enhance HCV treatment availability and accessibility for PWID are needed. Furthermore, linkage to care, follow-up, and harm reduction programs are critical to reducing HCV infection among PWID which is an ongoing challenge in Puerto Rico.

## Data Availability

The datasets generated and/or analyzed during the current study are not publicly available due to restrictions in which only authorized persons have access to the electronic NHBS database. To request more information, please contact the corresponding author.

## Abbreviations

Anti-HCV: Antibodies to hepatitis C virus
CDC: Centers for Disease Control and Prevention
HCV: Hepatitis C Virus
HIV: Human Immunodeficiency Virus
NHBS: National HIV Behavioral Surveillance System
PR-NHBS-PWID-5: Puerto Rico National HIV Behavioral Surveillance System PWID cycle five
PR: Puerto Rico
PWID: People Who Inject Drugs
RDS: Respondent-Driven Sampling
SJ-MSA: San Juan Metropolitan Statistical Area
STI: Sexually Transmitted Infection
UNODC: United Nations Office on Drugs and Crime
